# A prospective study of demographic features and quality of life in HIV-positive women with cervical cancer treated at Tygerberg Hospital

**DOI:** 10.4102/sajhivmed.v16i1.368

**Published:** 2015-09-18

**Authors:** George du Toit, Martin Kidd

**Affiliations:** 1 Department of Obsterics and Gynaecology, Stellenbosch University, South Africa; 2 Centre for Statistical Consultation, Stellenbosch University, South Africa

## Abstract

**Background:**

Cervical cancer and human immunodeficiency virus (HIV) infection/acquired immune deficiency syndrome (AIDS) both have a high incidence in South Africa. Cervical cancer treatment of HIV-positive women poses challenges. Treatment-related changes in quality of life (QOL) of such women are important to future treatment protocols.

**Aim:**

To examine demographic data of HIV-negative and HIV-positive women at diagnosis of cervical cancer and describe their changes in QOL as a result of treatment.

**Methods and materials:**

All newly diagnosed patients with cervical cancer at Tygerberg Hospital were approached to participate in the study. The European Organisation for Research and Treatment of Cancer Quality of Life Core Questionnaire (EORTC QLQ-C30) and the Cervix Cancer Module (QLQ-CX24) were used. General QOL was measured with the EORTC QLQ-C30 and cervical-specific QOL with the QLQ-CX24 questionnaire. The patients completed the questionnaire at diagnosis, on completion of treatment and at 3 months’ follow-up.

**Results:**

The study included a total of 221 women of whom 22% were HIV-positive; the latter were younger and of higher educational level than the rest. Mean monthly income and stage distribution was similar between the two groups. HIV-positive patients underwent radiation therapy more commonly than chemoradiation. HIV-positive women showed statistically significantly higher loss to follow-up during the study. HIV-positive women experienced no improvement in insomnia, appetite loss, nausea, vomiting, diarrhoea, social role or any of the sexual domains. In contrast, HIV-negative women experienced statistically significant improvement in all sexual domains other than sexual/vaginal functioning. The QOL improvement of HIV-negative women was statistically significantly greater than their HIV-positive counterparts in the majority of QOL domains. Global health improved in both groups, with HIV-negative women experiencing greater improvement. HIV-positive women experienced an initial decline of peripheral neuropathy (PN) symptoms post treatment with a return to pretreatment values at 3 months’ follow-up. The change in PN was statistically significant between the HIV-negative and HIV-positive women.

**Conclusion:**

Demographic differences exist between the HIV-negative and HIV-positive groups. The differential outcome in the QOL of HIV-positive and HIV-negative women treated for cervical cancer might be related to persistence of AIDS-related symptoms on completion of cervical cancer treatment.

## Introduction

The quality of life (QOL) of human immunodeficiency virus (HIV)-positive women with cervical cancer is the result of both diseases and the impact of their respective treatments. Invasive cervical cancer is an acquired immune deficiency syndrome (AIDS)-defining condition (World Health Organization stage 4).^1^ AIDS is endemic in sub-Saharan Africa. The South African population has a 12% – 18% incidence of HIV-positivity.^2^ South Africa has a cervical cancer incidence rate of 26.8/100 000.^3^ Most South African women present at an advanced stage of the disease. Cervical cancer and HIV infection are epidemiologically related owing to the sexual transmission of both conditions. Peripheral neuropathy (PN) in HIV-infected persons occurs in 50% – 60% of cases. At autopsy, PN can be shown in all HIV-positive persons despite their having no signs or symptoms during their lifetime. Antiretroviral medication (particularly didanosine, zalcitabine and stavudine) is directly neurotoxic and results in PN identical to AIDS-associated neuropathy. The disease and its treatment synergistically increase PN. Cisplatin is the drug of choice in chemoradiation (CR) treatment of cervical cancer. Cisplatin results, in a dose-dependent fashion, in sensory PN in the stocking-glove distribution.^4^ Poor tolerance of chemotherapy for cervical cancer by HIV-positive women results in substantially less completion of CR than their HIV-negative counterparts. The use of CR in advanced stage (III to IVA) cervical cancer in HIV-positive women has been questioned owing to the limited survival benefit.^5^ A Cochrane review shows a statistically non-significant 3% benefit in 5-year survival of CR over radiation therapy (RT) in stage III to IVA.^6^ Simonds et al.^5^ suggest that the omission of chemotherapy in these HIV-positive women with cervical cancer would result in timely completion of the full dose of radiation therapy.

A limitation of the study by Simonds et al.^5^ was the 15.4% (59 out of a cohort of 383) incidence of HIV-positive women.^5^ Data on the impact of RT on QOL of HIV-positive women with cervical cancer are lacking. The aim of the present study was to examine demographic data for HIV-negative and HIV-positive women at diagnosis of cervical cancer and to describe QOL changes in these women after treatment for cervical cancer.

## Methods and materials

### Inclusion criteria

Patients referred to the Unit of Gynaecologic Oncology at Tygerberg Hospital who had newly diagnosed cervical cancer were approached to participate in the study. The unit is one of two tertiary referral units for public-sector patients in Western Cape Province. The province has a population of 5.8 million. Most (85%) of the population do not have private medical insurance and are dependent on public facilities provided by two tertiary hospitals (Tygerberg Hospital and Groote Schuur Hospital) for treatment of cervical cancer.^7^ Patients were eligible for the study if they had histologically proven cervical cancer. Exclusion criteria included concurrent, or previous history of, cancers and medical disorders that might affect QOL, such as diabetes. Patients unable to provide informed consent owing to psychiatric disorders were excluded. Cervical cancer was staged according to international guidelines.^8^ Clinical management included HIV testing and initiation of antiretroviral treatment. HIV-positive women did not receive chemotherapy if their CD4 count was < 200 cells/µL, or active tuberculosis was present.

### Questionnaires

Patients completed the questionnaire in the language of their choice (isiXhosa, English or Afrikaans) after informed consent was obtained.^9^ A research assistant helped illiterate patients. To exclude bias, the research assistant had no medical background and was not involved in clinical management of the patients. Questionnaires were completed prior to treatment, after initial treatment, and after a 3-month post-treatment period. The follow-up visits coincided with clinical follow-up of patients. Patients failing to attend visits were contacted telephonically where possible. Patient records were used to extract relevant clinical data. Ethical approval was obtained from the local committee (S12/06/174). Clinical management followed protocols as previously described.^5^ The European Organisation for Research and Treatment of Cancer (EORTC) Quality of Life Core Questionnaire (EORTC QLQ-C30) and the Cervix Cancer Module (QLQ-CX24) were both used. The EORTC QLQ-C30 consists of 30 items comprising 5 functional scales (physical, role, emotional, social and cognitive), 3 symptom scales (fatigue, nausea/vomiting and pain), an overall QOL scale, and 6 individual items (dyspnoea, insomnia, appetite loss, constipation, diarrhoea, and financial difficulties). The EORTC QLQ-C30 was analysed according to the procedures recommended by the EORTC QOL Group. Higher scores on the QLQ-C30 functioning scales and the overall QOL scale indicate a better QOL. Higher scores on the symptom and individual item scales represent a decrease in QOL.^10^ The EORTC QLQ-CX24 includes 3 multi-item scales (symptom experience, body image, and sexual functioning) and 5 single-item scales (lymphoedema, lower back pain, menopausal symptoms, tingling and numbness, and sexual enjoyment). Higher scores indicate a decrease in QOL except for items 49 and 54 (where higher scores indicate better QOL).^11^ The questionnaires used were translated and validated for use in South Africa.^9^

### Statistical analysis

Descriptive statistics were used to characterise the study sample in terms of the contextual factors of socio-demographic and medical variables. Data presented as medians were analysed using Kruskal–Wallis tests. Post hoc analyses were done with Fisher's least significant difference (LSD) test. Chi-square tests were used for categorical data. A *p* value < 0.05 was considered to be significant. Statistical analysis was performed with the use of STATISTICA version 12 software.

## Results

### Demographic characteristics

The study included a total of 221 women (Table 1). HIV-positivity of the study group was 22%. The mean age of the HIV-positive women was statistically significantly 7 years less than that of the HIV-negative women. Age had a normal distribution without any outliers. HIV-positive women had a higher educational grade. Racial distribution shows a statistically significant difference between black (40%), mixed race (12%) and white (0%) participants’ HIV-positivity rates. Mean monthly income as well as the percentage of patients under the poverty line were not statistically significantly different between the HIV-positive and -negative groups. Single women had a statistically significantly higher rate of HIV-positivity than their married, widowed and divorced counterparts. The stage distribution of HIV-negative and HIV-positive cases was not statistically significantly different. HIV-positive patients underwent RT more commonly than CR.

**TABLE 1:** Comparative demographic data of HIV-negative and HIV-positive women (poverty line as defined by the Western Cape Provincial Government).

Characteristics	HIV-negative *n* = 173	HIV-positive *n* = 48	*p* value
**Age (years)**	51.34	43.94	*p* < 0.01
Mean education level (grade)	7	8	*p* < 0.05
**Race (%)**	-	-	*p* < 0.05
Mixed race people	88	12	-
Black people	60	40	-
White people	100	0	-
**Income (ZAR)**	1450	1576	NS
Below poverty line of R3500 (%)	79	21	NS
**Marital state (%)**	-	-	*p* < 0.05
Single	68	32	-
Married	88	12	-
Widow	86	14	-
Divorced	94	6	-
**Stage distribution**	-	-	NS
**Treatment (%)**	-	-	*p* < 0.05
Radiotherapy	75	25	-
Chemoradiation therapy	88	12	-
**Employment (%)**	-	-	*p* < 0.05
Employed	77	23	-
Pensioner	93	7	-
Unemployed	74	26	-

*Source*: Western Cape Provincial Treasury. Regional development profile City of Cape Town. 2012 [cited 2014 Jul 23]. Available from: http://www.westerncape.gov.za/assets/departments/treasury/dc0_city_of_cape_town_sep-lg_profile_02_2013.pdf

NS, not significant.

**FIGURE 1:**
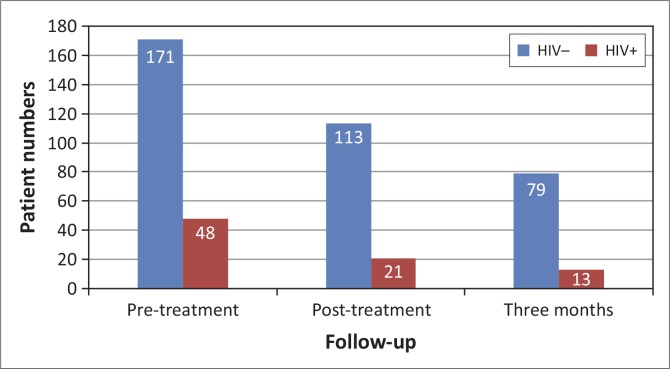
Follow-up of HIV-negative versus HIV-positive women over the study period.

Unemployed women had a statistically significantly higher HIV-positivity rate (26%) than the employed women (23%). The loss to follow-up of HIV-positive women v. HIV-negative women during the post-treatment (56% v. 34%) and 3-month (38% v. 30%) follow-up visits was statistically significantly higher for the HIV-positive women (Figure 1). Cause and confirmation of death could be accurately determined in 20 women in the total study population.

### HIV status and change in quality of life over the study period

The domains of dyspnoea, financial difficulties, lymphoedema and menopausal symptoms remained unchanged during the study period. HIV-positive women experienced no improvement in insomnia, appetite loss, nausea and vomiting, diarrhoea, social role or any of the sexual domains over the study period. In contrast, HIV-negative women experienced statistically significant improvement in all sexual domains other than sexual/vaginal function. The improvement in QOL of HIV-negative women was statistically significantly more than their HIV-positive counterparts in all domains, with the exception of role function, insomnia, constipation, sexual worry and sexual activity (Table 2). Global health improved in both groups, with HIV-negative women experiencing a greater improvement. PN did not change in HIV-negative women but HIV-positive women experienced an initial decline in this symptom at post treatment with a return to pretreatment values at the 3-month follow-up visit. The change in PN was statistically significantly different between HIV-negative and HIV-positive women.

**TABLE 2:** Change in quality of life during study period.

Quality of life domain	HIV- *n* = 173	HIV+ *n* = 48	HIV- versus HIV+
Physical function	*p* < 0.01†	*p* < 0.05†	*p* < 0.05
Role function	*p* < 0.01‡	*p* < 0.01‡	NS
Dyspnoea	NS	NS	NS
Pain	*p* < 0.01‡	*p* < 0.01‡	*p* < 0.01
Fatigue	*p* < 0.01‡	*p* < 0.01‡	*p* < 0.01
Insomnia	*p* < 0.01‡	NS	NS
Appetite loss	*p* < 0.01†	NS	*p* < 0.01
Nausea and vomiting	*p* < 0.01‡	NS	*p* < 0.01§
Constipation	*p* < 0.01†	*p* < 0.05†	NS
Diarrhoea	NS	NS	*p* < 0.01
Cognitive function	*p* < 0.01‡	*p* < 0.05‡	*p* < 0.01
Emotional role	*p* < 0.01‡	*p* < 0.01‡	*p* < 0.05
Social role	*p* < 0.01†	NS	*p* < 0.05
Financial difficulties	NS	NS	NS
Global health status	*p* < 0.01†	*p* < 0.01†	*p* < 0.01
Symptom experience	*p* < 0.01‡	*p* < 0.05‡	*p* < 0.01
Lymphoedema	NS	NS	NS
Peripheral neuropathy	NS	*p* < 0.01‡	*p* < 0.01
Menopausal symptoms	NS	NS	NS
Body image	*p* < 0.01†	*p* < 0.01†	*p* < 0.05
Sexual worry	*p* < 0.01‡	NS	NS
Sexual activity	*p* < 0.01†	NS	NS
Sexual/vaginal functioning	NS	NS	*p* < 0.01
Sexual enjoyment	*p* < 0.05†	NS	*p* < 0.01

NS, not significant.

†, Improved; ‡, decreased; §, HIV+ >HIV-.

All other *p* values in column HIV- >HIV+.

## Discussion

The results of the study show significant demographic differences between HIV-positive and HIV-negative women with a diagnosis of cervical cancer. The former group is statistically younger, and has a higher educational level and higher unemployment rate than the latter. Black women have a statistically higher HIV-positivity rate than mixed race and white women. Single women had the highest HIV-positivity rate. Monthly income is similar in both groups. RT was more frequently used than CR in HIV-positive patients. The 22% HIV-positive rate in the current study is higher than previously reported rates. This change is the result of a general change in HIV-positive rates in the total population over time.^2^ Black women had a higher HIV-positive rate than mixed race or white women. A previous study documented a higher incidence (30%) of positive syphilis serology amongst black women with cervical cancer than in their white and mixed race counterparts.^12^ The younger age of HIV-positive cervical cancer patients confirms previous studies of HIV in cervical cancer cases. In previous studies, the difference in mean age between HIV-negative and HIV-positive patients was reported as 10 years, whilst the current study shows a 7-year age difference.^5,13,14^ The stage distribution in the current study was similar in HIV-negative and HIV-positive women. Despite the similar stage distribution, significantly more HIV-negative than HIV-positive women received CR. The selection by the presiding clinician of the inability of HIV-positive women to tolerate the chemotherapy because of low CD4 counts, gave rise to this difference.

The majority of QOL domains in HIV-negative women improved with treatment with prolonged effect up to 3 months’ follow-up. Improvement of QOL domains in HIV-positive women was statistically less than in HIV-negative women. PN domain did not change in HIV-negative women. In HIV-positive women, initial improvement occurred in PN with relapse to pretreatment level at 3 months. Appetite loss in HIV-positive women initially improved after treatment and returned to pretreatment levels at 3 months’ follow-up. HIV-negative women showed an improvement in appetite loss up to 3 months’ follow-up. The QOL of HIV-negative women significantly improved in the majority of domains. HIV-positive women had fewer domains improved by treatment, and the magnitude of improvement was less than that amongst HIV-negative women. Temporary improvement of pain, fatigue and appetite loss after treatment in HIV-positive women reverted to pretreatment levels at 3 months’ follow-up. Pain and fatigue are AIDS-related conditions that are prevalent in AIDS patients, despite adequate treatment. Depression is associated with these symptoms, and the difference in emotional functioning in the current study underlines the element of depression in the HIV-positive women.^15^ The AIDS-related impact on QOL accounts for these relapses in QOL domains. Diarrhoea was significantly more in HIV-positive women than in HIV-negative women, and treatment did not change the incidence in either group. Diarrhoea is commonly associated with AIDS and can have numerous causes, both infectious and non-infectious, for example AIDS medication-related gastro-intestinal side-effects.^16^ Constipation improved in both HIV-negative and HIV-positive women. Radiation is associated with increased stool frequency owing to radiation-induced mucosal rectal damage. PN paradoxically improved in both groups after treatment and reverted to pretreatment levels in HIV-positive women. Contrary to expected cisplatin-related toxicity, treatment did not result in an increase of PN. The dose of cisplatin, which did not reach the cumulative threshold dose > 250 mg – 350 mg/m², may explain the absence of PN. Cisplatin-associated PN may occur up to 8 months after exposure, and therefore longer follow-up may reveal PN.^17^

In the present study, higher rates of loss to follow-up occurred in HIV-positive women. A meta-analysis of sub-Saharan low- and middle-income countries’ antiretroviral treatment programmes reports on causes of loss to follow-up. Self-transferring care to other facilities (18.6%), unreported death (38.8%) and stopping treatment were identified as the major reasons for loss to follow-up.^18^ WHO AIDS stage 3 and 4 cases have a mortality rate of 72.12 per 100 person-years in the first 6 months after initiation of treatment. The mortality rate decreases to 7.9 per 100 person-years after 12 months.^19^ The mortality rate is compounded by cervical cancer-related death. In a South African study, the mortality rate after treatment of stage III cervical cancer was the highest in the first 6 months after treatment.^12^ In Kenya, a 41% loss to follow-up occurred in women receiving treatment for cervical cancer.^20^ Tracking of women after missed appointments is not done routinely owing to resource constraints.^18^ Verifying HIV-related deaths by checking death certificates is subject to 90% misclassification of HIV deaths in South Africa.^21^

Limitations of the present study include a short follow-up subsequent to completion of therapy. The short follow-up limits the conclusion to long-term effects of treatment. Prolonged follow-up may reveal an increased incidence of PN. The higher loss to follow-up rate of HIV-positive women during the study period precludes sub-analysis of smaller groups, for example treatment-related PN in those women undergoing CR.

In conclusion, the study documents the demographic difference in HIV-negative and HIV-positive women with cervical cancer with regard to a younger age in the latter group. The 5-year survival benefit of CR in comparison with RT in HIV-negative women with stage III to IVA is a statistically non-significant 3%.^6^ The poor response of HIV-positive women to CR raises the question of whether CR is appropriate in these circumstances.^5^ A significant difference exists in the short term in certain QOL domains of HIV-positive women with cervical cancer receiving RT or CR. In these circumstances, the different impact on long-term QOL of HIV-positive women with cervical cancer receiving RT or CR warrants further study.
